# A Demand for State Aid: Conference at the Guildhall

**Published:** 1912-12-14

**Authors:** 


### RESEARCH WORK IN MENTAL HOSPITALS.

### A Demand for State Aid: Conference at the Guildhall.

An important conference of representatives of the Visiting Committees of Mental Hospitals in England and Wales was held at the Guildhall on Thursday last week. The object of the conference was to urge the need for State aid in the shape of grants towards research for the prevention, cure, and treatment of mental diseases.

The Lord Mayor of London, who presided at the outset, and who welcomed the delegates, said that they were greatly indebted to the Lord Mayor of Cardiff, who had initiated the conference and who had done so much in that great cause.

### The Presidential Address.

The Lord Mayor of Cardiff, Alderman Morgan Thomas, then took the chair and delivered his presidential address. He said that he would like to see the time arrive when all their asylums should be called mental hospitals. In view of the advanced state of mental science, and in order to assist the medical gentlemen who had charge of those institutions, they should do all that lay in their power to remove the idea that they were places merely of detention and not of cure. Their system of local government had delegated to the local authorities the administration of hospitals for the insane. He did not forget that there was a Treasury grant for the maintenance of the insane, but the chief burden for the erection of buildings and the care and maintenance of the patients fell upon the local authorities. If the work was to be a success throughout the country they looked to the medical staffs at each institution to throw themselves heart and soul into the work as they were doing at present. Grants were often given by the Local Government Board in other directions for research work, and there was no reason why it should not be done in the case of mental diseases. The Government had put aside a grant of something like a million and a-half of money to grapple with tuberculosis. But if anything a much sadder disease was the disease of insanity, where they had patients who could not convey intelligently how much they were suffering. It was really necessary that the medical staff, if it was to carry on its work successfully, should have every encouragement from the local authority.

### The Terms of the Resolution.

Sir George Savage moved the following resolution:—"That this meeting of representatives of the Visiting Committees of Asylums of England and Wales is of the opinion (*a*) that the prevention of insanity by hereditary transmission, and of acquired and recurring insanity, as also the cure of acquired insanity, are of prime importance to the State; (*b*) that it is expedient that State aid be given in the shape of grants towards specific scientific researches having for their object the prevention and cure of insanity, such grants to be made through the medium and upon the recommendation of His Majesty's Commissioners in Lunacy; that copies of the above resolution be forwarded to the Prime Minister, the Lord Chancellor, the Chancellor of the Exchequer, the Home Secretary, and the Commissioners in Lunacy."

He said that forty or fifty years ago the one consideration was to protect society from the dangerous lunatic. Gradually it became necessary more carefully to consider the lunatic rather than society apart from the lunatic. Many people had been in the habit of asking what was the use of the research work which their laboratories did. It was all important, however, that one should not look for the immediate results but for the possible and almost certain results in the future which could not be obtained sufficiently by isolated efforts.

### A Hall-mark for Research Workers.

Dr. J. Shaw Bolton, Superintendent of the West Riding Mental Hospital at Wakefield, who seconded the resolution, said that for many years insanity had been largely on the increase. There were more than 135,000 certified lunatics in the country, or one in 269 of the general population, and that number was increasing year by year. Over one-fourth of those admitted to the mental hospitals of this country had been insane previously, and over two-thirds of the persons admitted never left those institutions. Those facts were sufficient to show that were insanity any other form of disease it would be regarded by the medical profession and the public as an incurable affliction. Research had gradually rid them of the conviction that at any rate a large proportion of the types of insanity were either incurable or recurrent. While for almost a century efforts had been made to cure insanity, it was time that they should endeavour to take such steps as would prevent it. Such steps could only be taken as the result of research—whether chemical, physiological, psychological or biological. Research work was done for the benefit of the general public, and the general public should pay. Government grants would stimulate those engaged in the work, and would increase their sense of responsibility. The carrying out of research under those conditions would hall-mark the men who were at work.

Sir Thomas Crosby, President of the Bethlehem Hospital, said that the question might be asked whether the present means were not sufficient for research. The present means had utterly failed—he referred to the numerous post-mortem examinations of people who had died in mental hospitals. It was much better to be engaged in research work and to inquire into the causes of insanity in living people.

### The Mental Clinic.

Dr. James Soutar, President of the Medico-Psychological Association, said that while he accepted the main idea of the resolution before them, his experience made him hesitate to accept some of the subsidiary suggestions embodied therein. There were many competent observers, for instance, who contended that the influence of heredity had been somewhat overrated. If they believed in the full complete and absolute transmission by heredity they would prevent it in no other way than preventing the sufferers propagating their species. A large body of medical men were opposed to that view, and believed that an attempt on those lines would do infinitely more harm than good. He also urged that State aid should be given to the establishment of clinics for the treatment of incipient cases of mental disorder.

Sir Norval Helme, M.P., representing the Lancashire Asylums Board, said that the establishment of clinics as already in existence at Munich would be of immense value, for they would become receiving houses where people who feared a development of mental disease might go and receive scientific advice. He objected to the proposal that the grants should be made through the Commissioners in Lunacy, who were appointed by the Lord Chancellor and were not answerable to Parliament.

Alderman Benjamin Crowther (West Riding) said that the names workhouse and asylum had become obnoxious, and he would rather that they were described as hospitals for the poor and for the feeble-minded. He suggested that they might have one national centre for research provided entirely by the Government.

### A Plea for Decentralised Research.

Dr. McDowall, Medical Superintendent at the Northumberland County Mental Hospital, said that the Medico-Psychological Association had worked for the institution of a diploma in psychological medicine with the object of putting knowledge in the way of the young men working in their hospitals. Recently they had succeeded in persuading several universities to open courses of instruction for young medical officers whereby they might be able more thoroughly to train themselves for the very difficult work which they had to carry on. The Universities of Cambridge, Leeds, Manchester, Edinburgh, and Durham had instituted such a degree. The great difficulty in establishing clinics was that the public and the patients themselves would not recognise insanity in its earliest stages. Very few men were willing to admit it, and the clinics already established at various hospitals had been more or less unsuccessful for that reason. He would be very sorry to see only one central laboratory. They wanted research developed in every mental hospital and not merely at the centre.

Dr. Goodall (Cardiff) said that although a great deal of private support had been given to hospitals and universities in the founding of chairs and the promotion of research, none seemed to have come the way of the mental hospitals which had been put out of sight, and therefore out of mind. There was also a feeling that such institutions were on the rates which could support anything.

### The Motion that was Carried.

After further discussion the original resolution was withdrawn, and the following motion was carried unanimously at the suggestion of Sir Norval Helme:—"That it is expedient that State aid be given in the shape of grants towards research for the prevention, cure, and treatment of mental diseases, and that copies of this resolution be sent to the Prime Minister, the Lord Chancellor, the Chancellor of the Exchequer, the Home Secretary, and the Commissioners in Lunacy."

A deputation was appointed to wait upon the Lunacy Commissioners and the Home Secretary, consisting of the Lord Mayor of Cardiff, Sir George Savage, Dr. Shaw Bolton, Sir Thomas Crosby, Dr. Goodall, Dr. W. Hodgson, Dr. James Soutar, Alderman Benjamin Crowther, Sir Norval Helme, M.P., and Sir G. W. Truscott.

